# The exposure of autoantigens by microparticles underlies the formation of potent inflammatory components: the microparticle-associated immune complexes

**DOI:** 10.1002/emmm.201201846

**Published:** 2012-12-11

**Authors:** Nathalie Cloutier, Sisareuth Tan, Luc H Boudreau, Catriona Cramb, Roopashree Subbaiah, Lauren Lahey, Alexandra Albert, Ruslan Shnayder, Reuben Gobezie, Peter A Nigrovic, Richard W Farndale, William H Robinson, Alain Brisson, David M Lee, Eric Boilard

**Affiliations:** 1 Faculté de Médecine de l'Université Laval, Centre de Recherche en Rhumatologie et Immunologie, Centre de Recherche du Centre Hospitalier Universitaire de QuébecQuébec, Québec, Canada; 2 Molecular Imaging and NanoBioTechnology, IECB, UMR-CBMN University Bordeaux-1Pessac, France; 3 Division of Immunology and Rheumatology, Department of Medicine, Stanford University School of MedicineStanford, CA, USA; 4 Department of Orthopaedics, Case Western Reserve University, School of MedicineCleveland, OH, USA; 5 Division of Rheumatology, Immunology and Allergy, Brigham and Women's Hospital, Harvard Medical SchoolBoston, MA, USA; 6 The Cleveland Shoulder Institute, University Hospitals of ClevelandCleveland, OH, USA; 7 Division of Immunology, Boston Children's Hospital, Harvard Medical SchoolBoston, MA, USA; 8 Department of Biochemistry, University of CambridgeDowning Site, Cambridge, UK; 9 Novartis Institutes for Biomedical ResearchBasel, Switzerland

**Keywords:** arthritis, autoantigens, immune complexes, microparticles, platelets

## Abstract

Immunoglobulins, antigens and complement can assemble to form immune complexes (IC). ICs can be detrimental as they propagate inflammation in autoimmune diseases. Like ICs, submicron extracellular vesicles termed microparticles (MP) are present in the synovial fluid from patients affected with autoimmune arthritis. We examined MPs in rheumatoid arthritis (RA) using high sensitivity flow cytometry and electron microscopy. We find that the MPs in RA synovial fluid are highly heterogeneous in size. The observed larger MPs were in fact MP-containing ICs (mpICs) and account for the majority of the detectable ICs. These mpICs frequently express the integrin CD41, consistent with platelet origin. Despite expression of the Fc receptor FcγRIIa by platelet-derived MPs, we find that the mpICs form independently of this receptor. Rather, mpICs display autoantigens vimentin and fibrinogen, and recognition of these targets by anti-citrullinated peptide antibodies contributes to the production of mpICs. Functionally, platelet mpICs are highly pro-inflammatory, eliciting leukotriene production by neutrophils. Taken together, our data suggest a unique role for platelet MPs as autoantigen-expressing elements capable of perpetuating formation of inflammatory ICs.

## INTRODUCTION

Upon activation, cells of many different lineages bud small extracellular vesicles called microparticles (MP) (also known as microvesicles or ectosomes) from the cytoplasmic membrane. Given the universality of this process, MPs likely play essential functions that include cell–cell communication and transportation of mediators (Beyer & Pisetsky, 2010; Distler et al, 2005b; Gyorgy et al, 2011b).

It is increasingly appreciated that elevated levels of MPs are evident in diverse diseases (Beyer & Pisetsky, 2010; Distler et al, 2005b; Gyorgy et al, 2011b). Platelet MPs in particular were observed in autoimmune disorders including systemic lupus erythematosus (SLE; Pereira et al, 2006; Sellam et al, 2009), Sjögren's syndrome (Sellam et al, 2009), multiple sclerosis (Sheremata et al, 2008), antiphospholipid syndrome (Jy et al, 2007), and RA (Boilard et al, 2010, 2012; Knijff-Dutmer et al, 2002; Sellam et al, 2009). Interestingly, platelets are potently capable of releasing MPs. Indeed, platelet MPs were the first type of MPs to be described originally designated as “platelet dust” found abundantly in plasma (Chargaff & West, 1946; Wolf, 1967).

Immune complexes (IC) represent immunoglobulins and complement clustered about target antigen. They are extremely potent at eliciting inflammatory responses and implicated in response to infections as well as multiple autoimmune diseases such as Sjögren's syndrome (Voulgarelis & Tzioufas, 2010), SLE (Munoz et al, 2010), multiple sclerosis (Cross et al, 2001; Ziemssen & Ziemssen, 2005), antiphospholipid syndrome (Ruiz-Irastorza et al, 2010) and RA (Nigrovic, 2006). Given the simultaneous presence of ICs and MPs in autoimmune disorders, we examined MPs keeping in mind that they could themselves serve as antigenic surface for formation of functional ICs, referred to hereafter as microparticle ICs (mpICs).

Using high sensitivity flow cytometry (hs-FCM), transmission electron microscopy and proteomic approaches, we found that ICs in RA synovial fluid frequently include MPs, thereby forming true mpICs. Although we observed that MPs originating from platelets express FcγRIIa, mpICs formed not through binding to this receptor but rather by recognition of citrullinated autoantigens such as vimentin and fibrinogen. Together, these results identify a unique site of autoantigen expression in RA and suggest that MPs, including those derived from platelets, may participate in the generation of a novel type of potent ICs in this disease.

## RESULTS

### MPs in SF are highly heterogeneous in size

MPs are widely recognized as ∼100–1000 nm extracellular vesicles that can exhibit phosphatidylserine. These uncommon features permit the detection of MPs cytofluorometrically by employing Annexin-V (which recognizes phosphatidylserine) conjugated to fluorochromes and size discrimination (Connor et al, 2010; Gyorgy et al, 2011a; Perez-Pujol et al, 2007; Thiagarajan & Tait, 1991). However, standard flow cytometry is not well adapted to the detection of such small MPs. The demonstration of the common biophysical properties of ICs and MPs (Gyorgy et al, 2011a) and the recent development of improved flow cytometry approaches for MP detection prompted us to further analyse MPs in RA.

**Figure 1:**
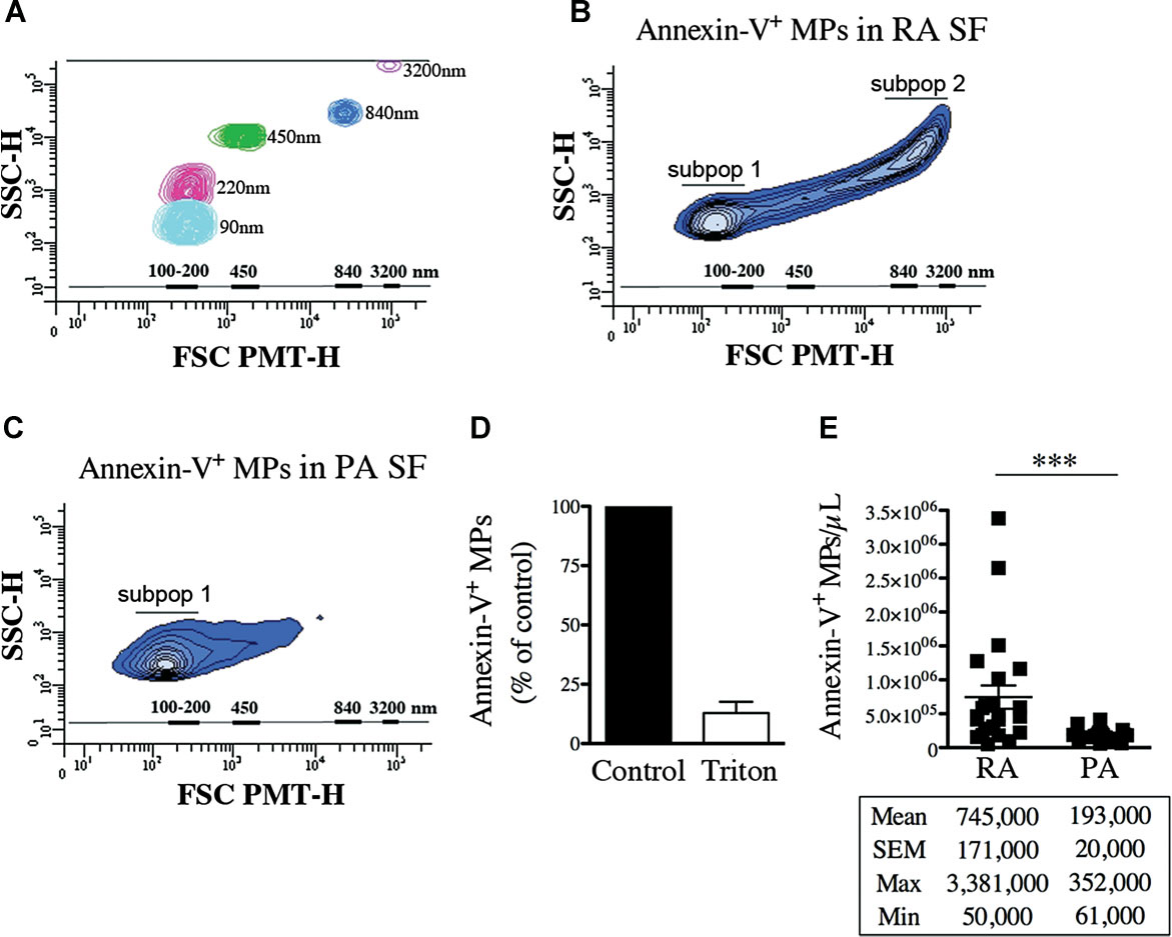
MPs are heterogeneous in size and are more abundant in RA compared to PA SF **A.** Acquisition of fluorescent Sky Blue microspheres of 40–90 nm (mean = 90 nm, in cyan blue), 100–300 nm (mean = 220 nm, in pink), 400–600 nm (mean = 450 nm, in green), 700–900 nm (mean = 840 nm, in blue), 2500–4500 nm (mean = 3200 nm, in violet) diameter on a flow cytometer Canto II modified with a FSC-PMT small particles option. A scale bar ranging from 100 to 3200 nm based on the microsphere sizes (FSC-PMT) is presented and used to determine the relative dimensions of the MPs.**B,C.** Representative FSC-PMT and SSC portrayals of the Annexin-V^+^ events detected in RA SF (**B**) and PA SF (**C**) revealing the dimension diversity of the MPs. The relative dimensions of the MPs are presented according to size-defined microsphere calibrations. Two major subpopulations (subpop 1 and 2) are detected in RA SF (**B**) while only one (subpop 1) is observed in PA SF (**C**) are identified on the graphs.**D.** Triton sensitivity of the MPs in RA SF detected using Annexin-V labeling presented as % of untreated (control).**E.** Flow cytometric quantifications of the Annexin-V^+^ MPs contained in RA and PA SF (*n* = 23 RA and *n* = 18 PA ***p = 0.0004). Statistical analyses are presented under the graph. Data are mean ± SEM.

After optimizing high sensitivity flow cytometry (hs-FCM) parameters to distinguish small particles (Fig 1A), we assessed size and quantity of MPs in RA. For comparison, the MPs present in psoriatic arthritis (PA) – a disease family not associated with intra-articular ICs and complement consumption – were also examined. Similarly to MPs, ICs can display dimensions ranging from 100 to 1000 nm (Gyorgy et al, 2011a). Further, since it is hypothesized that ICs can interact with multiple MPs to form larger components, we included all particles up to 3500 nm in diameter for our analyses. Interestingly, unlike MPs in PA SF, MPs in RA SF appear highly heterogeneous in size and include two major subpopulations, one exhibiting diameters ranging from ∼100 to ∼300 nm and a second one from ∼700 to ∼3000 nm (Fig 1B and C).

Given the common biophysical properties of ICs and MPs and the prominent quantities of ICs in SF (Gyorgy et al, 2011a), we utilized an established detergent lysis protocol to differentiate these entities (Gyorgy et al, 2011a). With this method, membrane phospholipids from MPs are solubilized by detergent while the ICs remain intact (Gyorgy et al, 2011a). We observe that the Annexin-V^+^ particles are promptly solubilized by Triton X-100 treatment, a finding consistent with the membrane composition present in MPs (Fig 1D). These analyses reveal 745,000 ± 171,000 Annexin-V^+^ MPs/µl in SF of RA patients (*n* = 23). For comparison, we find 193,000 ± 20,000 Annexin-V^+^ MPs/µl in SF of PA patients (*n* = 18), significantly lower than the concentrations detected in RA (*p* = 0.0004; Fig 1E). Interestingly, we observe that MPs in RA SF are not only more abundant than in PA SF, they also display different dimensions, suggesting that they are associated with other macromolecular structures.

### MPs and ICs associate in SF from patients with RA, not PA

**Figure 2:**
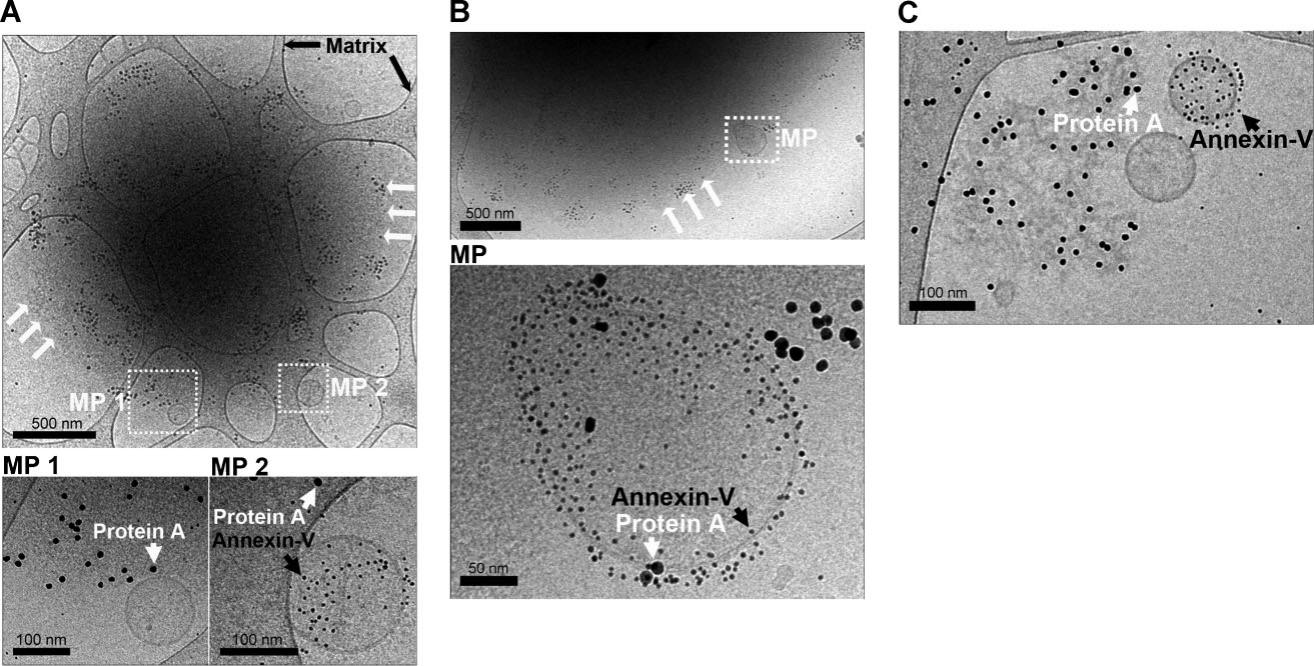
Visualization of the mpICs in RA SF Cryo-TEM detection of immunoglobulins and phosphatidylserine exposing MPs using, respectively protein A-conjugated gold nanoparticles (10 nm) and Annexin-V-conjugated gold nanoparticles (4 nm). **A,B.** Representative observations obtained using RA SF in which MPs [white boxes labelled MP (MP 1 and MP 2 (A) and MP (B)] were colocalized with ∼2 µm diameter mpICs. White arrows indicate the edge of the IC where MPs were detected. Black arrows indicate the carbon matrix of the perforated support film used for TEM. Insets (bottom panels) include individual MPs. Immunoglobulins detected using protein A conjugated nanospheres (10 nm) are indicated with white arrowheads and MPs detected using Annexin-V conjugated nanospheres (4 nm) are indicated by black arrowheads. Note that some MPs were Annexin-V negative.**C.** Relatively smaller mpICs were also visualized. White and black arrowheads indicate immunoglobulins and phosphatidylserine, respectively. Scale bars are presented under each panel.

Intrigued by the presence of large dimension MPs in RA SF, we hypothesized that MPs could bind immunoglobulins and thereby form large MP-containing ICs (mpICs). To assess the presence of mpICs in RA SF, we used cryo-TEM, which allows direct visualization of small particles and their membrane bilayers. We further examined the exposure of phosphatidylserine in RA SF mpICs using Annexin-V conjugated to 4 nm gold nanoparticles and identified immunoglobulin containing ICs using larger gold nanoparticles (10 nm) conjugated to protein A. Using this method, we identify macromolecular structures up to 2 µm in diameter that contain both ICs and MPs (mpICs). Interestingly, these mpICs often harbour multiple MPs at the periphery (Fig 2A–C), ∼65% of them expressing phosphatidylserine. Importantly, these observations were verified in freshly obtained RA SF (*n* = 5), ruling out the involvement of freezing–thawing in an artifactual generation of mpICs.

**Figure 3:**
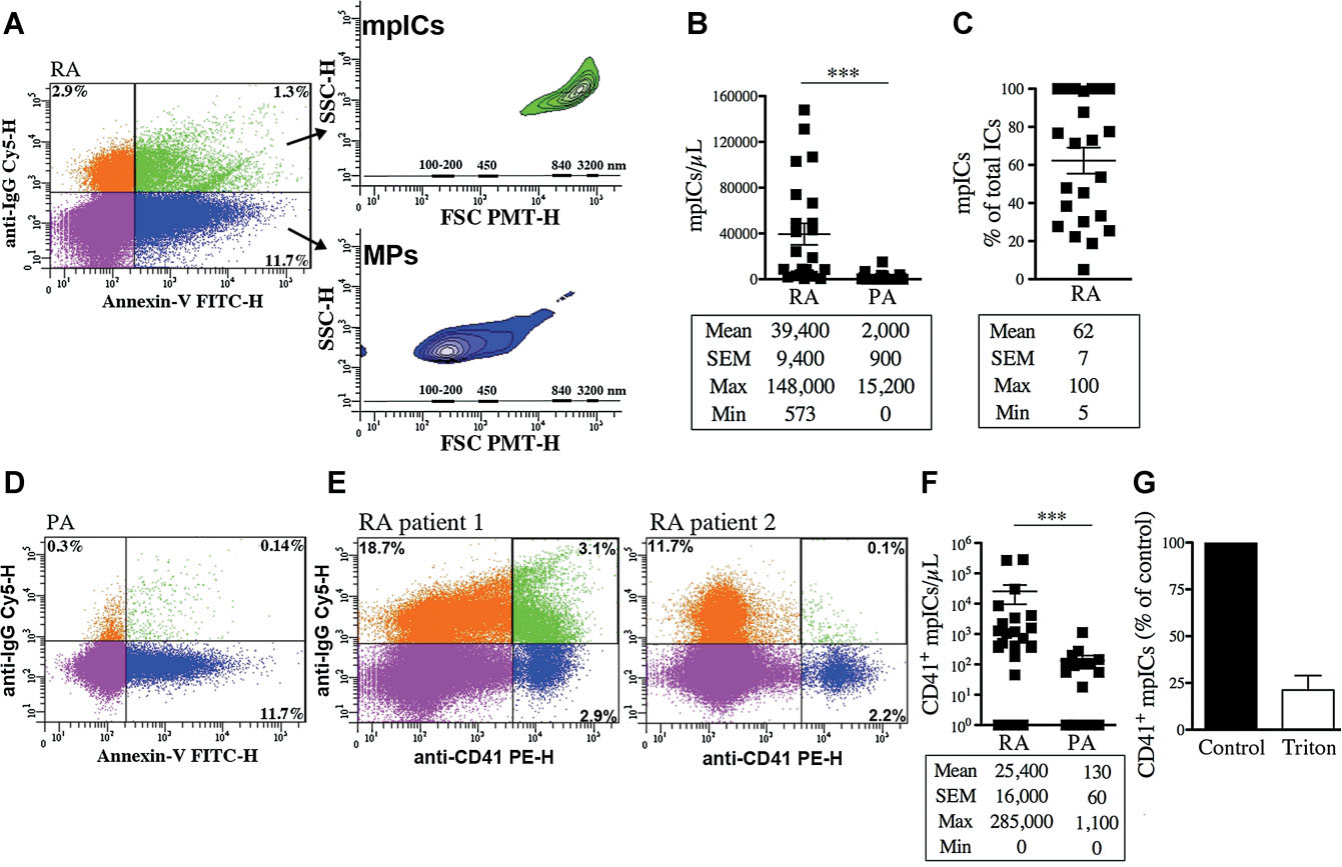
MPs, including platelet MPs, in RA SF form mpICs FSC-PMT and SSC dot plots of representative RA SF labelled with a combination of FITC-conjugated Annexin-V and Cy5-conjugated anti-IgG to demonstrate the presence of IgG on surface of Annexin-V^+^ MPs. The four-quadrant gates were positioned according to the isotypic controls. The MPs (Annexin-V^+^-IgG^−^) in blue show dimensions ranging mostly from 100 to 300 nm (lower inset) while the mpICs (Annexin-V^+^-IgG^+^) in green have dimensions from 700 to 3000 nm (upper inset). The relative diameters are presented according to size-defined microsphere calibrations.Quantifications of the mpICs contained in RA and PA SF (*n* = 23 RA and *n* = 18 PA ***p < 0.0001).Quantifications of the mpICs relative to the total amounts of detectable ICs in RA SF (*n* = 23). The statistical analyses are presented under each graph. Data are mean ± SEM.FSC-PMT and SSC dot plots of representative PA SF labelled with a combination of FITC-conjugated Annexin-V and Cy5-conjugated anti-IgG.FSC-PMT and SSC dot plots of RA SF patients labelled with PE-conjugated anti-CD41 and Cy5-conjugated anti-IgG. The four-quadrant gates were positioned according to the isotypic controls. The presence of CD41^+^ mpICs is evidenced by the dual expression of CD41 and IgG by MPs (green region). RA SF from two patients are presented to illustrate the heterogeneity that exists among the patients.Quantifications of the CD41^+^ mpICs in RA and PA SF (*n* = 25 RA and *n* = 18 PA ***p = 0.0006). The statistical analyses are presented under each graph. Data are mean ± SEM.Triton sensitivity of the CD41^+^ mpICs contained in RA SF presented as % of untreated (control) CD41^+^ mpICs.

Having visualized mpICs, we employed hs-FCM to analyse them (Fig 3A). We measure 39,400 ± 9400 mpICs/µl in RA SF (Fig 3B). The quantification of the ICs in these same fluids (Supporting Information Fig S1) reveals that the majority (62 ± 7%) of the detectable ICs are in fact mpICs (Fig 3C). These observations provide an explanation for the presence of two subpopulations of MPs evident in RA SF. Specifically larger particles (from ∼700 to 3000 nm; upper inset in Fig 3A) contain mpICs. Conversely, the MPs not associated with IgG are characterized by smaller dimensions (100–300 nm; lower inset in Fig 3A) and represent the majority of the total Annexin-V^+^ MPs (93% ± 1.4; Supporting Information Fig S2). Interestingly, although MPs and IgG are both present in PA SF, only 2000 ± 900 mpICs/µl could be detected (Fig 3B and D).

### Platelet-derived mpICs are present in RA SF

Having established the presence of mpICs in RA SF, we next investigated mechanisms by which ICs and MPs combine. A key question for further mechanistic investigation is identification of the cellular source(s) of MPs in SF. Considering their role in arthritis (Boilard et al, 2010, 2012), we queried whether platelet MPs contribute significantly to mpICs in RA SF. For these analyses, we assessed the presence of the platelet specific integrin CD41 in mpICs using hs-FCM. Consistent with the vast heterogeneity that prevails among RA patients, the amount of CD41^+^ mpICs in RA SF differs from one patient to another (*n* = 25; two examples shown on Fig 3E). Interestingly, the number of CD41^+^ mpICs in RA SF is significantly higher than those observed in PA SF (*n* = 18; *p* = 0.0006; Fig 3F). Importantly, although the ICs remain intact after detergent treatment, the CD41^+^ MPs contained in mpICs are detergent soluble, further establishing their phospholipid composition (Fig 3G).

### Platelet mpICs form independently of FcγRIIA

**Figure 4:**
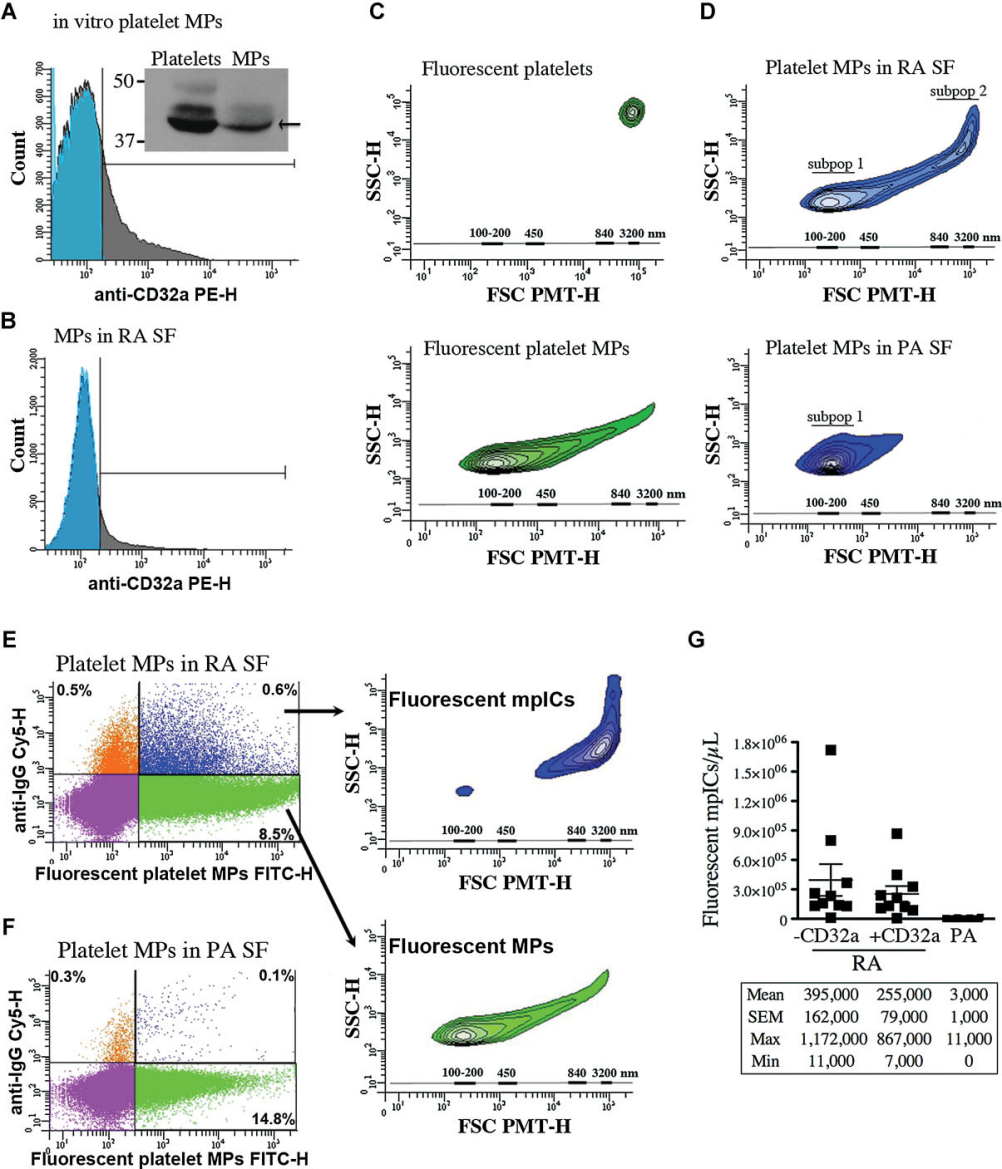
Platelet MPs form mpICs independently of CD32a **A,B.** The expression of CD32a by platelet MPs was determined by hs-FCM and Western blot analysis (inset in **A**). The platelet MPs were generated *in vitro* upon platelet GPVI stimulation (**A**) and the endogenous MPs in RA SF were detected using Annexin-V (**B**). Blue: isotype; grey: specific CD32a labeling (*n* = 3).**C.** FSC-PMT and SSC profiles of the fluorescent platelets (top panel) and platelet MPs (bottom panel) (*n* = 10). The relative dimensions are presented according to size-defined microsphere calibrations.**D–G.** Results obtained following the incubation of exogenous fluorescent platelet MPs in RA and PA SF (*n* = 10 RA and *n* = 18 PA). (**D**) Representative FSC-PMT and SSC plots obtained using RA (top) and PA SF (bottom). Two subpopulations (subpop) (1 and 2) were detected in RA and only one in PA SF (1). (**E**,**F**) hs-FCM evaluation of the presence of IgG on surface of MPs upon incubation in RA (**E**) and PA SF (**F**) using Cy5-conjugated anti-IgG antibody. The four-quadrant gates were positioned according to the isotypic controls. (**E**) MPs (in green) are IgG^−^ and show dimensions ranging from 100 to 300 nm (lower inset) while the mpICs (in blue) have dimensions ranging from 700 to 3000 nm (upper inset). (**D**,**E**) The relative dimensions are presented according to size-defined microsphere calibrations. (**G**) hs-FCM quantifications of the mpICs were determined in absence (1st and 3rd column) or presence (2nd column) of CD32a blocking antibody (1st and 2nd column *p* = 0.4478 (unpaired *t*-test), 1st and 3rd column ***p < 0.0001). Statistical analyses are presented under the graphs. Data are mean ± SEM.

Human platelets express the IC receptor FcγRIIA (CD32a; Huang et al, 2011; Parren et al, 1992). We thus postulated that CD32a present on platelet-derived MPs may contribute to formation of mpICs. In this set of experiments, we initially assessed CD32a expression by platelet-derived MPs. Since platelets promptly release MPs following stimulation of the collagen receptor glycoprotein VI (GPVI) (Boilard et al, 2010; Knight et al, 1999), and since GPVI deficiency in mice leads to reduction of the severity of arthritis (Boilard et al, 2010), we generated MPs via this arthritis-relevant stimulus. Here, we find that platelet-derived MPs retain CD32a (Fig 4A). Examination of MPs in RA SF demonstrates they too harbour CD32a (Fig 4B).

To assess a contribution of platelet MP CD32a in mpIC formation, we incubated *in vitro* generated platelet MPs in the presence of RA SF (*n* = 10) or PA SF (*n* = 18). In this assay, the fluorescent label present in *in vitro* MPs affords discrimination of newly formed mpICs from those present endogenously. As anticipated, the fluorescent platelet MPs are easily detected by hs-FCM, are smaller than intact platelets, the vast majority of them having dimensions of ∼100–300 nm in diameter (Fig 4C). Strikingly, the incubation of fluorescent platelet MPs in RA SF yields two subpopulations (Fig 4D), including the larger dimension (700–3000 nm) subpopulation that contains mpICs (Fig 4E). Interestingly, this subpopulation does not form when the platelet MPs are exposed to PA SF (Fig 4D, F and G). To explore the contribution of CD32a in formation of mpICs in RA SF, we pre-incubated the platelet MPs with a blocking CD32a antibody. We find that blockade of CD32a does not impede formation of mpIC (Fig 4G; *n* = 10, *p* = 0.4478).

### Role of citrullination in generation of mpICs

**Figure 5:**
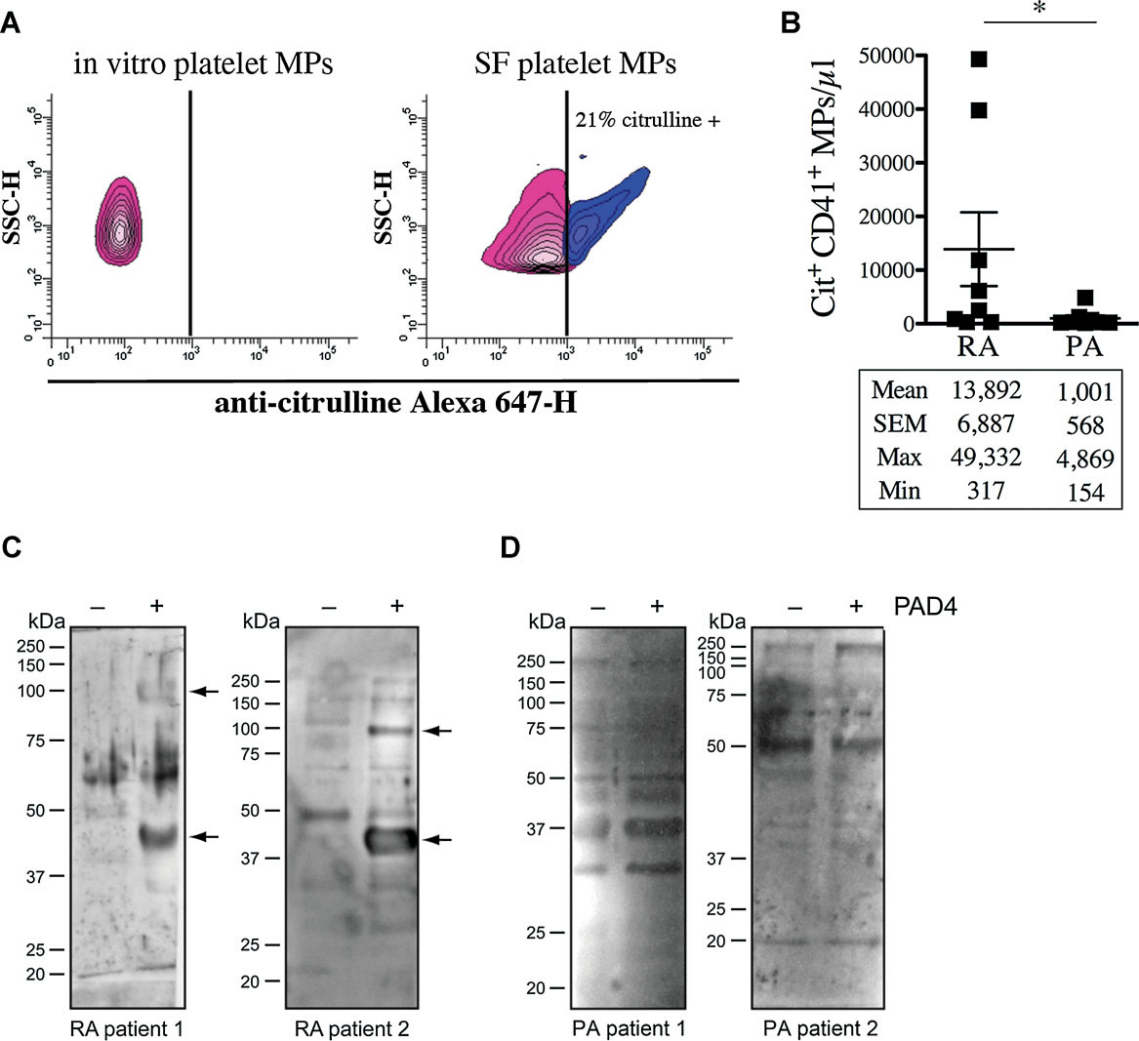
Platelet MPs display citrullinated autoantigens recognized by IgG from RA SF **A,B** The presence of citrullinated epitopes on platelet MPs was determined by hs-FCM using anti-citrulline antibody and its corresponding secondary antibody conjugated to Alexa 647. MPs were confirmed of platelet origin using a PE-conjugated anti-CD41 antibody. (**A**) The presence of citrullinated antigens on platelet MPs was determined in *in vitro* platelet MPs (left panel) and MPs from RA SF (right panel). The vertical lines were positioned according to the isotypic controls. Pink: Negative population; blue: Positive population. The % of positive platelet MPs is indicated on graphs. (**B**) Quantification of the citrulline^+^ CD41^+^ MPs in RA and PA SF (*n* = 8 RA and *n* = 8 PA *p = 0.0148).**C,D** The proteins from platelet MPs obtained *in vitro* left unmodified (−PAD4) or citrullinated (+PAD4) were separated on SDS–PAGE and transferred to membranes. The membranes were incubated in presence of RA (**C**) or PA (**D**) SF and the recognition of MP-derived autoantigens by autoantibodies from SF was revealed using HRP-conjugated anti-human IgG. Arrows indicate proteins recognized by IgG from SF uniquely when the MPs were citrullinated. Two RA and PA SF are presented to illustrate the existing variability between patients (*n* = 12 RA and *n* = 8 PA).

Autoantibody/antigen binding comprises another plausible mechanism for formation of mpIC. Among the mechanisms that occur in RA SF and not in PA SF is citrullination, a process that generates neoepitopes recognized by autoantibodies present in abundance in many patients with RA (Yoshida et al, 2006). Specifically, citrullination is a posttranslational modification of peptidyl-arginine to peptidyl-citrulline by peptidyl arginine deiminase (PAD) enzymes; in RA SF, the PAD4 isoform levels are significantly elevated (Arita et al, 2004; Lundberg et al, 2005). Given that MPs are present in a milieu where citrullination is active, we hypothesized that the MP surface proteins may become citrullinated and give rise to autoantigens recognized by autoantibodies in RA patients and thereby promote mpIC formation. Using hs-FCM, we first evaluated whether platelet MPs are citrullinated in the course of RA pathogenesis. We find that, unlike platelet MPs generated *in vitro* and those in PA SF, the platelet MPs in RA SF contain citrullinated epitopes (Fig 5A and B), pointing to citrullination of MPs as a potential mechanism leading to recognition by autoantibodies.

To evaluate the contribution of citrullination on MPs to recognition by autoantibodies in RA SF, we incubated platelet MPs in presence of purified PAD4 and assessed their antigenicity to IgG from RA SF. Interestingly, we find that multiple citrullinated proteins in platelet MPs are recognized by autoantibodies from RA patients (6 RA patients out of 12; 2 patients represented in Fig 5C). For comparison, we evaluated whether antibodies from PA could recognize citrullinated proteins from platelet MPs. We demonstrate that citrullination of MPs does not increase binding of antibodies from SF of PA patients (0 PA patient out of 8 tested; 2 patients represented in Fig 5D), consistent with the minimal presence of mpICs in this disease (Fig 3B and F).

### Identification of the autoantibodies derived from mpICs

**Figure 6:**
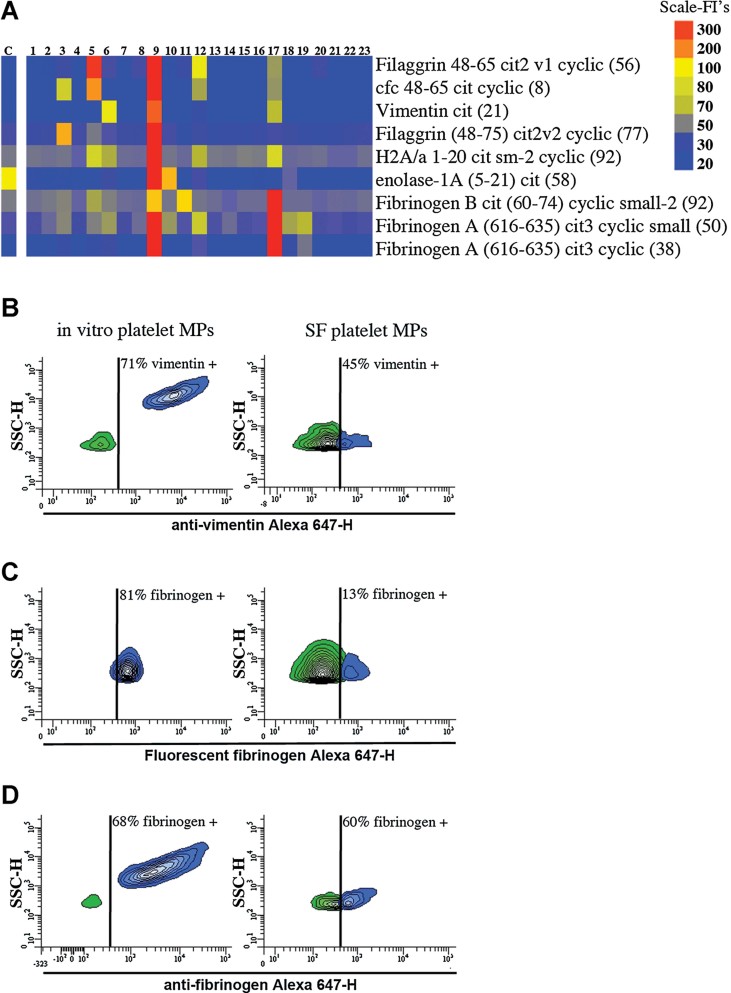
Platelet MPs in RA SF exhibit autoantigens **A.** Identification of the autoantibodies contained in CD41^+^ mpICs in RA SF. The binding specificity of the mpIC-eluted IgG to 40 antigens was determined on a Bio-Plex™ bead-based antigen array and only the antigens that presented significant binding are presented (*n* = 23 RA patients, identified 1–23). Colours denote amount of binding: undetectable (blue); progressively greater binding (green, yellow, red). Color scale is presented on right. The negative control (C) was obtained by incubating isotypic antibodies in SF. Cfc; cyclic citrullinated filaggrin, H2A/a; histone H2A/a.**B–D.** The presence of autoantigens was examined on *in vitro* platelet MPs (left) and platelet MPs from RA SF (right) by hs-FCM (*n* = 4). (**B**) MPs were incubated with anti-vimentin and the interaction was revealed using Alexa 647-conjugated secondary antibody. (**C**) MPs were incubated with exogenous Alexa 647-fluorescent fibrinogen and associations quantified by hs-FCM. (**D**) MPs were incubated with anti-fibrinogen and the interaction with endogenous fibrinogen was determined using Alexa 647-conjugated secondary antibody. The vertical lines were positioned according to the isotypic controls. Green: negative population; blue: positive population. The % of positive MPs is indicated on graphs.

Proteomic analyses permit the identification of the antigens targeted by autoantibodies (Hueber et al, 2005; Monach et al, 2009; Zhao et al, 2008). To identify autoantigens expressed by platelet MPs, we isolated the CD41^+^ mpICs from RA SF using magnetic affinity columns. These CD41^+^-enriched preparations, composed of immunoglobulins and complement C3a (466.6 ± 193 pg C3a/µg protein, *n* = 23), were applied to protein G affinity columns and the IgG within mpICs were isolated. Using an autoantigen microarray (Hueber et al, 2005; Robinson et al, 2002), we demonstrate that the IgG from platelet mpICs associate with a broad series of recognized citrullinated autoantigens (Fig 6A).

### MPs exhibit autoantigens

Having identified the autoantibodies in platelet mpICs, we next used mass spectrometry to determine whether platelet mpICs also include autoantigens. We demonstrate that several canonical RA autoantigens are present in platelet mpICs (Table 1). Among these autoantigens present in platelet mpICs and recognized by the IgG eluted from platelet mpICs, we focused our attention on the two previously described autoantigens vimentin (El-Gabalawy & Wilkins, 2004; Kurki et al, 1983) and fibrinogen (Takizawa et al, 2006). Vimentin is an intracellular cytoskeleton protein expressed on the surface of platelet-derived MPs (Podor et al, 2002) and autoantibodies directed against citrullinated-vimentin are present in RA SF (El-Gabalawy & Wilkins, 2004; Kurki et al, 1983). We thus examined the expression of vimentin on surface of *in vitro* generated platelet-derived MPs and platelet MPs contained in RA SF. We find that the surface of both *in vitro* generated platelet MPs and those isolated from RA SF exhibit significant expression of vimentin (Fig 6B).

**Table 1:** Identification of the autoantigens included in CD41^+^ MPs from RA SF

Protein ID	Number of peptides^a^
Apolipoprotein A-1	7; 8; 4; 5
Clusterin	0; 2; 0; 0
Fibrinogen alpha chain	6; 16; 14; 11
Fibrinogen beta chain	0; 13; 15; 6
Histone H2A	2; 3; 3; 3
Histone H2B	2; 4; 2; 4
Vimentin	0; 1; 0; 2

a The number of peptides detected for each protein is indicated for each RA patient.

Fibrinogen is also a known autoantigen in RA and autoantibodies directed against citrullinated fibrinogen suffice to induce arthritis in mice (Ho et al, 2010). While fibrinogen is a ligand of the platelet integrins GPIIb/IIIa (Bodary et al, 1989), we confirmed that fibrinogen interacts with platelet MPs. We therefore examined the ability of platelet MPs to associate with fluorescent fibrinogen using hs-FCM. Interestingly, exogenous fibrinogen promptly binds to platelet MPs obtained *in vitro* and to platelet MPs from RA SF, demonstrating that MPs of platelet origin associate with the autoantigen (Fig 6C). Fibrinogen is expressed in the platelet's alpha granules (Sehgal & Storrie, 2007), is present in RA SF (Takizawa et al, 2006) as well as being an abundant plasma protein. We thus evaluated whether platelet MPs interacted with endogenous fibrinogen *in vitro* and *ex vivo*. Interestingly, we demonstrate that, similar to platelet MPs produced *in vitro*, platelet MPs present in RA SF exhibit surface fibrinogen (Fig 6D). Together, these data show that platelet MPs are capable of displaying autoantigens vimentin and fibrinogen which, when citrullinated, could allow binding by autoantibodies in RA SF.

### Platelet mpICs are pro-inflammatory

**Figure 7:**
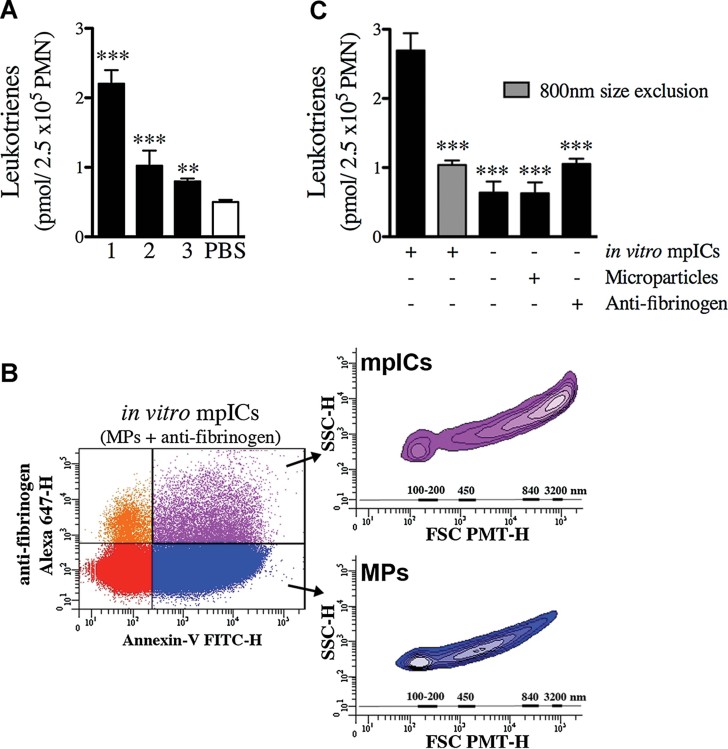
Platelet mpICs promote leukotriene production by neutrophils Quantifications of the leukotriene biosynthesis by neutrophils incubated in presence of RA SF mpICs [*n* = 3 distinct patients ***p < 0.0001 patients 1 and 2; **p = 0.0011 patient 3 (unpaired *t*-test, relative to PBS control)]. Data are mean ± SEM. Leukotrienes measured represent the sum of LTB4, 6-trans-LTB4, 12-epi-6-trans-LTB4, 20-OH-LTB4 and 20-COOH-LTB4.Platelet mpICs form promptly upon co-incubation of platelet MPs with anti-fibrinogen *in vitro*. Representative FSC-PMT and SSC dot plot of mpICs. The presence of mpICs formed *in vitro* was demonstrated using a combination of FITC-conjugated Annexin-V and Alexa 647-conjugated secondary antibody. The four-quadrant gates were positioned according to the isotypic controls. The MPs (Annexin-V^+^-IgG^−^) presented in blue show dimensions ranging mostly from 100 to 500 nm (lower inset) while the mpICs (Annexin-V^+^-IgG^+^) presented in purple display dimensions ranging from 800 to 3000 nm (upper inset). The relative dimensions are presented according to size-defined microsphere calibrations.Quantifications of the leukotriene biosynthesis by neutrophils incubated in presence of *in vitro* mpICs, anti-fibrinogen and platelet MPs. The stimulatory potency of mpICs compared to the others conditions was statistically significant [*n* = 3 ***p ≤ 0.0001 (unpaired *t*-test)]. Data are mean ± SEM. Leukotrienes measured represent the sum of LTB4, 6-trans-LTB4, 12-epi-6-trans-LTB4, 20-OH-LTB4 and 20-COOH-LTB4.

The inflammatory lipid mediators leukotrienes are present in RA synovial fluid (Klickstein et al, 1980) and its production by neutrophils, a cellular lineage dominant in the synovial fluid from RA patients (Edwards & Hallett, 1997), contribute to the pathophysiology of inflammatory arthritis in *in vivo* mechanistic models (Chen et al, 2006). Similarly to MPs, the mpICs can be centrifuged and used as cellular stimulus. We thus centrifuged SF from RA patients and used the resulting pellet to determine its potency on human neutrophils. We demonstrate that this fraction, which contains both MPs and mpICs, can stimulate neutrophils to produce leukotrienes (Fig 7A). We next used *in vitro* generated mpICs to examine whether they could elicit the release of leukotrienes from neutrophils. We find that mpICs, which spontaneously form after the co-incubation of platelet MPs with anti-fibrinogen (Fig 7B), induce robust leukotriene production by neutrophils (Fig 7C). For comparison, platelet MPs and anti-fibrinogen are only modestly active when added to neutrophils separately (Fig 7C). To assess the relative pro-inflammatory activity of mpICs, we fractionated mpICs, MPs and anti-fibrinogen ICs via size exclusion filtration (800 nm pores). Interestingly, we show that the mpIC depletion abrogates much of the leukotriene stimulation in this assay (Fig 7C), establishing that the platelet mpICs are active and highly pro-inflammatory.

## DISCUSSION

Our work identifies a highly pro-inflammatory macromolecular structure composed of MPs and immune complexes abundant in RA synovial fluid. Further, our mechanistic studies show that MPs, including platelet MPs, in RA SF are not naked phospholipid vesicles. Rather, they express or associate with autoantigens, can be citrullinated and form bioactive inflammatory mpICs.

The identification of MPs as a site of autoantigen expression extends previous seminal observations regarding MPs and ICs in RA synovial fluid. Others had suggested that complement and immunoglobulins were associated with the substantial majority of MPs in RA SF (Biro et al, 2007). However, no membrane markers such as Annexin-V, or lineage markers such as CD41, were utilized to distinguish MPs from ICs in those standard flow cytometry experiments. More recent demonstration of common biophysical properties (size, light scattering, sedimentation) between ICs and MPs has called into question whether or not MPs and immunoglobulins are associated in RA SF and demonstrate the need for using lineage markers or other methods to further clarify the interpretation of previous observations (Gyorgy et al, 2011a). Our findings refine and unify these discrepant observations by demonstrating that although only a proportion (∼7%) of the MPs are part of mpICs, the majority of the detectable ICs are in fact mpICs.

The presence of immunoglobulins on surface of MPs in blood during RA has been previously examined (Nielsen et al, 2012; van Eijk et al, 2010). In contrast to our demonstration of MP interactions with ICs in RA SF, those studies did not find evidence of MP association with ICs in RA blood. Although these observations may point to the existence of highly potent vascular and reticular-endothelial system clearance mechanisms, this may also suggest that the mpICs form locally, inside the inflamed joint. Indeed, the MPs derived by platelets in blood during RA (Knijff-Dutmer et al, 2002; Sellam et al, 2009) may accumulate in the SF through the gaps present between the endothelial cells in the inflamed vasculature (Boilard et al, 2012; Cloutier et al, 2012). Once in SF (where PAD4 is present), MPs may undergo citrullination and form mpICs. The exact anatomical site where mpICs form and the mechanism(s) of transportation of mpICs remains the subject of intense investigation.

Although the detection of phosphatidylserine is commonly used to determine the presence of MPs, Annexin-V^−^ MPs are also observed in substantial numbers associated with ICs. Nevertheless, all the mpICs we examined by cryo-TEM were composed of at least one Annexin-V^+^ MP, suggesting that quantifications of mpICs based on Annexin-V detection still remain reasonably accurate. Although little is known about the origin of these Annexin-V^−^ MPs, they are also abundant in plasma (Connor et al, 2010) and may originate from distinct stimuli. Indeed, platelet activation via collagen or thrombin produces Annexin-V^−^ MPs (Connor et al, 2010; Perez-Pujol et al, 2007). Their presence provides evidence for further significant diversity of MPs in RA SF.

While platelet-derived MPs do express CD32a, its contribution to formation of mpICs is quantitatively modest. On the other hand, we show that platelet MPs can interact with autoantibodies against citrullinated antigens via at least two mechanisms, (i) following the citrullination of its surface and (ii) upon binding to citrullinated proteins. Autoantigens other than vimentin and fibrinogen may also be expressed on surface of platelet MPs during RA. Indeed, multiple citrullinated proteins derived from PAD4-treated MPs are recognized by RA autoantibodies. Further, we also identify autoantibodies against canonical citrullinated RA autoantigens such as filaggrin (including cfc), enolase and histone H2A/a in mpICs. Among these other proteins, only histones were also identified in our mass spectrometry analyses. Histones may be associated with DNA, itself bound to MPs. Alternatively, histones are recognized potent platelet stimuli and can associate with platelets directly (Fuchs et al, 2011). Platelet MPs, like platelets, may thus interact with histones and could be recognized by anti-histone autoantibodies. Although the contribution of these autoantigens to mpIC formation remains to be identified, we establish that major autoantigens in RA are exposed on the surface of platelet-derived MPs.

Other types of extracellular vesicles may express autoantigens. Small apoptotic CD3^+^ T cells, for instance, expose vimentin (Boilard et al, 2003) and could potentially interact with anti-vimentin autoantibodies if present in RA SF. Furthermore, exosomes, vesicles derived from intracellular compartments, also display antigens in RA SF (Skriner et al, 2006). MPs from other cellular origins (other than platelet-derived) may also express autoantigens. Considering that MPs from multiple sources populate the SF (Berckmans et al, 2002; Boilard et al, 2010), it is highly plausible that they too form mpICs. We revealed the capability of PAD4 to use platelet MPs as substrate. This process is likely to occur on other MPs, regardless of their origin, promoting the formation of additional citrullinated autoantigens in RA SF. However their identity and contribution remain to be established.

Recognizing the pro-inflammatory functions of MPs (Berckmans et al, 2005; Boilard et al, 2010; Distler & Distler, 2010; Distler et al, 2005a; Messer et al, 2009; Reich et al, 2011) and IC (Ho et al, 2010; Korganow et al, 1999; Kuhn et al, 2006; Stuart & Dixon, 1983) when taken separately, our identification of mpICs in synovial fluid uncovers a previously unappreciated physiology of potential relevance both to host defense and to autoimmune disease. During infections, ICs can efficiently trigger the opsonization and the clearance of pathogens. Platelets are highly potent producers of MPs and there is a growing appreciation of the role of platelets in immunity (Boilard et al, 2012; Semple et al, 2011). Although the recognition of bacterial products by platelets can initiate the production of MPs (Brown & McIntyre, 2011), whether the formation of inflammatory mpICs can occur in a physiologically beneficial manner in the course of the responses against pathogens is unknown.

In the context of autoimmunity, we show that platelet MPs can support immunoglobulin deposition. Since platelet MPs are abundant in autoimmune diseases (Berckmans et al, 2005; Boilard et al, 2010; Distler & Distler, 2010; Distler et al, 2005a; Messer et al, 2009; Reich et al, 2011), they may be a crucial source of autoantigens for the constant autoantibody deposition that perpetuates inflammation in chronic disorders. We further demonstrate that platelet mpICs are highly potent in promoting the production of leukotrienes by neutrophils. In addition to their capability to activate leukocytes, mpICs may harbour unique roles, not played by conventional ICs. Although we do not exclude that MPs also nucleate ICs, our analyses permitted us to visualize MPs at the periphery of the ICs. An inflammatory mpIC may thus interact with different cell types or may be directed to specific locations via the adhesion molecules present on the MPs that populate its surface. Further, we speculate that the formation of mpICs contributes to disease pathophysiology in other disorders. ICs are also present in multiple inflammatory autoimmune disorders and are reported to propagate disease (Cross et al, 2001; Ho et al, 2010; Korganow et al, 1999; Kuhn et al, 2006; Monach et al, 2009; Munoz et al, 2010; Ruiz-Irastorza et al, 2010; Stuart & Dixon, 1983; Voulgarelis & Tzioufas, 2010; Zhao et al, 2008; Ziemssen & Ziemssen, 2005). Indeed, circulating MPs in SLE patients are associated with autoantibodies and complement (Nielsen et al, 2012; Ullal et al, 2011). In sum, the mechanism behind the creation of functional mpICs, presented here, may well apply to other biological contexts.

In conclusion, we report that in RA, platelet MPs exhibit antigens that are recognized by RA specific autoantibodies. ICs are commonly described as immunoglobulins-, antigens- and complement-containing structures. We clearly establish that ICs can also contain MPs, including platelet MPs, and form pro-inflammatory mpICs. These observations extend previous definitions about the nature of ICs and expand our insights into the contributions of MPs and ICs to disease physiology in RA. Considering the rapidly emerging functions of inflammatory MPs, our findings point to potential roles in diverse autoimmune diseases characterized by presence of autoantibodies and MPs.

## MATERIALS AND METHODS

### Collection and preparation of synovial fluids

Human knee SFs as discarded material from patients with various arthritides undergoing diagnostic by the treating attending rheumatologist or therapeutic arthrocentesis. All studies received Institutional Review Board approval (BWH). Fresh SF harvested without anticoagulant were cleared of leukocytes by centrifugation at 1900*g* for 30 min at 4°C, were aliquoted and were kept at −80°C.

### Platelet MP production and isolation

Platelets were obtained from citrated blood of healthy human volunteers under an approved Institutional Review Board protocol (CRCHUQ, Université Laval). Platelets were isolated (Boilard et al, 2011), resuspended at 100 × 10^6^/ml in Tyrode's buffer and stimulated using 0.5 µg/ml cross-linked collagen related peptide (Kehrel et al, 1998) for 120 min at RT. The activation reaction was stopped by addition of 20 mM EDTA. The preparation was centrifuged at 1300*g* for 5 min twice, then at 18,000*g* for 90 min at 18°C and the pellet containing the MPs was resuspended in Tyrode's buffer. When fluorescent MPs were required, platelets were incubated in presence of 5 µM Cell Tracker CMFDA (5-chloromethylfluorescein diacetate, Invitrogen, ON, Canada) for 15 min prior to stimulation.

### Flow cytometry analyses of MPs and pre-analytical steps

MPs contained in SF were not pelleted before labelling to ensure that most of the MPs we analysed (Lacroix et al, 2012). MPs were not filtered to ensure we analysed the larger mpICs. All of the buffers were filtered on 0.2 µm pore size membranes (Fisher Scientific, ON, Canada). A forward scatter (FSC) coupled to a photomultiplier tube (PMT) ‘small particles option’ (FSC-PMT) (rather than the usual diode; van der Vlist et al, 2012) with a 488 nm solid state, 100 mW output blue laser (rather than the conventional 20 mW), a 633 nm HeNe, 20 mW output red laser and a 405 nm solid state diode, 50 mW output violet laser were mounted on the FACS Canto II Special Order Research Product used for all our studies (BD Biosciences, ON, Canada). The chosen parameters were optimal to detect particles from 100 to 3500 nm simultaneously on the FSC-PMT. In all experiments, the sensitivity and specificity of the MP labelling were rendered optimal by using fluorochromes excited by three distinct lasers.

### MP and IC labelling

SF (10 µl) were incubated with 0.5 µg of Cy5-conjugated F(ab′)_2_ goat anti-human IgG (anti-IgG-Cy5) (Jackson ImmunoResearch, PA, USA) and 0.5 µg of PE-conjugated mouse anti-human CD41 (M148) (anti-CD41-PE) (Abcam, MA, USA) in combination to 5 µl of FITC or V450-conjugated Annexin-V (BD). The samples were labelled during 30 min in a volume of 100 µl of Annexin-V buffer 1× and diluted to 600 µl before hs-FCM analysis. To process the data quantitatively, 100,000 polystyrene microsphere (15 µm diameter; Polysciences, PA, USA) were added to each tube and 1000 microspheres were acquired. The included Annexin-V^+^ or CD41^+^ events were portrayed as FSC-PMT *versus* side scatter (SSC) graph and the relative dimensions were displayed according to the acquisition of Sky Blue microspheres of mean diameter of 90, 220, 450, 840 and 3200 nm (Spherotech, IL, USA). The sensitivity of the MPs to detergent was assessed by addition of Triton X-100 to the SF samples (1:5 in PBS) prior to addition of the antibodies (Gyorgy et al, 2011a).

### IgG–MP interaction

Fluorescent MPs (20 × 10^6^) were incubated in presence of 50 µl RA or PA SF in final volume 100 µl PBS for 48 h at 4°C. Then, 50 µl of the cocktail were incubated with anti-IgG-Cy5 as described above. When the contribution of CD32a was evaluated, 20 µg/ml of CD32a blocking Fab fragment antibody (clone IV.3, gift from Dr Paul Naccache, Université Laval) was added 15 min prior to MP addition to SF.

### Western blotting

Platelets and MPs were prepared as described above, lysed and the protein content was determined using the Pierce BCA protein assay kit (Fisher Scientific). One microgram of sample was lysed in Laemmli buffer, electrophoresed, transferred onto membranes and incubated in 0.2% milk/TBS-Tween solution containing a serum raised against a peptide in the cytoplasmic domain of CD32a (CT10; Ibarrola et al, 1997). The membrane was washed, treated with Peroxidase-AffiniPure anti-rabbit-IgG (Jackson ImmunoResearch) and reactive proteins were visualized by chemiluminescence (Perkin Elmer, MA, USA).

### Evaluation of presence of antigens on MP surface

Fluorescent platelet MPs (5 × 10^6^) or RA SF (5 µl pre-incubated with Annexin-V-V450) were incubated with 2 µl of PE-conjugated anti-human CD32 (clone IV.3) (Beckman Coulter, ON, Canada). To determine the capability of MPs to associate with exogenous fibrinogen, 15 µg/ml of Alexa Fluor-647-conjugated fibrinogen (Invitrogen) was incubated with 10 × 10^6^ platelet MPs or added to 10 µl of RA SF in a final volume of 100 µl for 48 h at 4°C. Their associations were evaluated using Cell-Tracker fluorescence (for platelet MPs) or anti-CD41-PE (for SF). To detect vimentin and fibrinogen on surface of MPs, platelet MPs (20 × 10^6^) and RA SF (20 µl) were pre-incubated with 20 µg/ml of CD32a blocking Fab fragment antibody for 15 min to abrogate any risk of potential non-specific binding of primary/secondary antibodies to platelet Fc receptor. MPs were then incubated with 25 µg/ml goat anti-fibrinogen (Abcam) or 1:100 goat anti-human vimentin (Sigma–Aldrich, ON, Canada) for 48 h at 4°C. Samples were washed, centrifuged and pellets retrieved in 100 µl of PBS. For flow cytometry detection, 6.25 µg of DyLight 649-conjugated F(ab)′_2_ anti-goat IgG (Jackson ImmunoResearch) and anti-CD41-PE were added.

### *In vitro* citrullination

Platelet MPs were prepared as described above and citrullinated *in vitro* by rabbit muscle peptidyl arginine deiminase (PAD, Sigma–Aldrich). 350 × 10^6^ MPs were incubated with/without 3 U of PAD for 18 h as described (Kuhn et al, 2006). Samples were lysed in Laemmli buffer and processed as described above. The PVDF membranes were incubated 18 h in 10% of SF diluted in TBS-Tween, washed and treated with Peroxidase-AffiniPure Goat Anti-Human IgG (Jackson ImmunoResearch).

### IgG BioPlex

To isolate the IgG contained in CD41^+^ mpICs, RA SF (300 µl) were incubated in presence of 1.2 µg of anti-CD41-PE for 30 min, then with 50 µl anti-PE magnetic beads (Miltenyi Biotec (MACS), CA, USA) for 20 min at RT and diluted to 1 ml in PBS. The preparations were applied to LS magnetic MACS columns, washed with 3 ml PBS 3 times and eluted with 4.5 ml PBS. The eluted CD41^+^ MPs were centrifuged at 18,000*g* for 90 min at 18°C and pellets retrieved in 100 µl of PBS containing 0.5% Triton X-100 and 0.5% NP40. The CD41^+^ MP lysates were applied to a G-protein column, washed with 7 ml PBS, then with 2 ml PBS pH 5.0 and the IgG eluted with 100 mM glycine pH 2.5. The pH of the fraction was adjusted to 7.4 and conserved at −80°C. The reactivity of the antibodies retrieved from the mpICs against forty candidate RA autoantigens (see Supporting Information Table S1 for detailed list) were determined using a custom Bio-Plex™ bead-based antigen array as previously described (Hueber et al, 2005; Monach et al, 2009; Zhao et al, 2008). Candidate RA peptide antigens were coupled to spectrally distinct beads using *N*-hydroxysuccinimide ester chemistry. Peptides were synthesized with a C-terminal biotin and coupled to streptavidin-coated beads. The beads were pooled and incubated with mpICs-derived IgG at RT. The remainder of the assay was performed as previously described (Sokolove et al, 2012) and the scores calculated. For comparison, a negative control where the synovial fluid was incubated with an isotypic antibody was included. Proteins were considered positive if the average fluorescence intensity of the RA MPs was greater than twice that of the control. Results were displayed in Java TreeView® (Version 1.1.3; Eisen et al, 1998).

**The paper explained**

PROBLEM:

Immune complexes (ICs) are formed after antibody deposition against antigens and propagate inflammation in autoantibody-driven autoimmune diseases. Extracellular vesicles of submicron dimensions, called microparticles, are also often present in the same pathologies where ICs are observed. Understanding how ICs continuously form is of high relevance to human diseases.

RESULTS:

We used a new generation of flow cytometer and sophisticated cryo-transmission electron microscopy approaches to characterize and quantify ICs and microparticles in the synovial fluid from patients affected with rheumatoid arthritis (RA). We identify a new class of ICs that predominates in RA; the microparticle-associated immune complexes (mpICs). We further reveal that platelet microparticles participate to the formation of mpICs via exposure of the canonical RA autoantigens. We demonstrate that these platelet mpICs are highly active, eliciting pro-inflammatory leukotriene release by neutrophils.

IMPACT:

Considering the abundance of platelet microparticles in autoimmune disease such as RA, this suggests that microparticles may serve to the constant autoantibody deposition and perpetuation of inflammation in chronic autoimmune inflammatory disorders.

### Cryo-TEM and gold labelling of mpICs from RA SF

For cryo-Transmission Electron Microscopy (TEM) experiments, samples were diluted 10× in a buffer containing 150 mM NaCl, 2 mM CaCl_2_ and 10 mM HEPES, pH 7.4. A 10 µl aliquot of SF was mixed with 1 µl of 10 nm Protein A-conjugated gold nanoparticles (Ted Pella, Inc.; 0.5 µg/ml final concentration), incubated for 30 min, mixed with 1 µl of 4 nm Annexin-V-conjugated gold nanoparticles suspension (1.4 × 10^15^ particles/L final concentration) and further incubated for 15 min. Aliquots (4 µl) were deposited on TEM grids coated with a perforated carbon film and quickly frozen in liquid ethane. TEM grids were mounted on a Gatan 626 cryoholder and transferred into a Tecnai F20 (FEI) microscope operated at 200 kV. Images were recorded with an USC1000-SSCCD camera (Gatan). The synthesis and characterization of Annexin-V-conjugated gold nanoparticles will be presented elsewhere (patent application Mornet and Brisson WO2007122259).

### Identification of autoantigens expressed by platelet MPs from RA SF

We used mass spectrometry to survey the presence of known autoantigens in platelet MPs in RA SF (see Supporting Information Table S2 for detailed list). Protein concentration from individual SF CD41^+^ MPs (isolated using MACS as described above) was measured using Dc Protein Assay (Bio-Rad, Hercules, CA). Twenty-five micrograms proteins from individual SF CD41^+^ MP sample were reduced and alkylated by using 21 mM triethyl phosphine (TEP) and 58 mM iodoethanol (IE). The samples were speed dried in a high speed vacuum centrifuge and redissolved in 100 µl of 0.1 M ABC buffer containing 0.1% perflurooctanoic acid (PFOA). Trypsin was added in the ratio of 1 µg per 50 µg of proteins. The samples were placed in a 37°C water bath for 16 h. The reaction was stopped by drying the samples and adding 70% IPA in ammonium hydroxide (50 mM) solution, pH 10. The enzyme was inactivated by reducing (TEP/acetonitrile) at 45°C for 1 h and then alkylating (iodoethanol/CH3CN) in the dark at 45°C for 2 h. A 1:1:1 solution of ethanol, ethyl acetate and HPLC grade water with 0.1% TFA was added to the dried peptide digest and subjected to high speed vacuum centrifuge to remove PFOA. The samples were then analysed by LC–MS/MS. A total of four SF biological replicates were analysed by mass spectrometry. PFOA was purchased from TCI America (Portland, OR, USA). All other reagents were of the highest quality commercially available.

### Mass spectrometry and data analysis

The protein digests were analysed by LC–MS/MS using an Ultimate 3000 LC system (Dionex, San Francisco, CA) interfaced to an LTQ-Orbitrap XL mass spectrometer (Thermo-Finnigan, Bremen, Germany). The platform operated in the nano-LC mode using the standard nano-ESI API stack fitted with a picotip emitter (uncoated fitting, 10 µm spray orifice, New Objective, Inc., Woburn, MA). The solvent flow rate through the column was maintained at 300 nl/min using a 1:1000 splitter system. The protein digests (5 µl) were injected into a reverse-phase C18 PepMap trapping column (0.3 × 5 mm^2^, 5 µm particle size, Dionex, Inc.) equilibrated with 0.1% formic acid/2% acetonitrile v/v and washed for 5 min with the equilibration solvent at a flow rate of 25 µl/min, using an isocratic loading pump operated through an auto-sampler. After the washing step, the trapping column was switched in-line with a reversed-phase C18 Acclaim PepMap 100 column (0.075 × 150 mm^2^, Dionex, Inc.) and the peptides were chromatographed using a linear gradient of acetonitrile from 4.8 to 40% in aqueous 0.1% formic acid over a period of 96 min at the above-mentioned flow rate such that the eluate was directly introduced to the mass spectrometer. An 80% acetonitrile elution step was subsequently performed for 5 min prior to resetting the analytical column to the initial equilibration conditions for 11 additional minutes at the end of the chromatographic run. The mass spectrometer was operated in a data-dependent MS to MS/MS switching mode, with the five most intense ions in each MS scan subjected to MS/MS analysis. The full scan was performed at 60,000 resolution in the Orbitrap detector and the MS/MS fragmentation scans were performed in the ion trap detector (IT) CID mode such that the total scan cycle frequency was approximately 1 s. The threshold intensity for the MS/MS trigger was always set at 1000 and the fragmentation was carried out using the CID mode using normalized collision energy (NCE) of 35. The data was collected in the profile mode for the full scan and centroid mode for the MS/MS scans. The dynamic exclusion function for previously selected precursor ions was enabled during the analysis such that the following parameters were applied: repeat count of 2, repeat duration of 45 s, exclusion duration of 60 s and exclusion size list of 150. Xcalibur software (version 2.0.7), Thermo-Finnigan, Inc., San Jose, CA) was used for instrument control, data acquisition, and data processing. MS raw data files were searched against Swiss-Prot (version 57) database using Mascot database search software (version 2.1.04, Matrix Science, London, UK) to identify peptides/proteins with the following parameters: S-hydroxyethylation of cysteine as a fixed modification, and oxidation of methionine to methionine sulfoxide as variable modifications. The mass tolerance for the precursor ion was set to 10 ppm, and for the product ion it was set to 0.8 Da. Strict trypsin specificity was applied, allowing for one missed cleavage. Peptides with a minimum score of 20 were considered as significant.

### Isolation and stimulation of neutrophil and leukotriene biosynthesis

Human neutrophils isolation and leukotriene quantifications were determined as described (Boyum, 1968; Flamand et al, 2002). Neutrophils were incubated at 37°C for 30 min in presence of RA SF mpICs, platelet MPs (1 × 10^9^ MPs/ml), anti-fibrinogen (50 µg/ml), or *in vitro* mpICs. RA SF mpICs were enriched by centrifugations of 100 µl of RA SF (18,000*g* for 90 min at 18°C) and resuspended in 10 µl of PBS. *In vitro* mpICs were generated by pre-incubation of MPs (1 × 10^9^/ml) and goat anti-fibrinogen from Abcam (50 µg/ml) at RT for 30 min. Reactions were stopped by addition of 500 µl cold methanol/acetonitrile (1:1) containing 12.5 ng of internal standards PGB_2_ and 19-OH-PGB_2_. Samples were stored at −20°C O/N then analysed by reverse-phase HPLC as previously described (Borgeat et al, 1990).

### Statistical analysis

Statistical significance was determined using non-parametric Mann-Whitney test. When indicated, unpaired Student's *t*-test was employed. All the statistical analyses were done using Prism software package 4.00 (GraphPad Software, CA, USA).

## Author contributions

NC: study conception, study design, acquisition of data, analyses and interpretation of data, manuscript preparation, statistical analyses; ST: acquisition of data, analyses and interpretation of data, manuscript preparation; LHB: study design, acquisition of data, analyses and interpretation of data, manuscript preparation, statistical analyses; CC: acquisition of data, manuscript preparation, statistical analyses; RSu: acquisition of data, manuscript preparation, statistical analyses; LL: acquisition of data, manuscript preparation, statistical analyses; AA: acquisition of data, analyses and interpretation of data; RSh: acquisition of data, analyses and interpretation of data; RG: interpretation of data, manuscript preparation; PAN: contributed critical reagent, analyses and interpretation of data, manuscript preparation; RWF: generated critical reagent, manuscript preparation; WHR: analyses and interpretation of data, manuscript preparation; AB: acquisition of data, analyses and interpretation of data, manuscript preparation; DML: study conception, analyses and interpretation of data, manuscript preparation; EB: study conception, study design, acquisition of data, analyses and interpretation of data, manuscript preparation.

## Supplementary material

As a service to our authors and readers, this journal provides supporting information supplied by the authors. Such materials are peer reviewed and may be re-organized for online delivery, but are not copy-edited or typeset. Technical support issues arising from supporting information (other than missing files) should be addressed to the authors.
