# Supplementary material for: The exposure of autoantigens by microparticles underlies the formation of potent inflammatory components: the microparticle-associated immune complexes
Source: EMBO Mol Med. 2012 Dec 11;5(2):235–49. doi: 10.1002/emmm.201201846 (PMC3569640; doi:10.1002/emmm.201201846)
Supplement: Supplementary file 1 [file emmm0005-0235-SD1.pdf]

EMBO Molecular Medicine

The exposure of autoantigens by microparticles underlies the formation of potent inflammatory components: the microparticle-associated immune complexes

Nathalie Cloutier, Sisareuth Tan, Luc H. Boudreau, Catriona Cramb, Roopashree Subbaiah, Lauren Lahey, Alexandra Albert, Ruslan Shnayder, Reuben Gobezie, Peter A. Nigrovic, Richard W. Farndale, William H. Robinson, Alain Brisson, David M. Lee and Eric Boilard

Corresponding author: Eric Boilard, CHUQ Research Center

Review timeline:

Submission date:	08 August 2012
Editorial Decision:	15 September 2012
Revision received:	17 October 2012
Editorial Decision:	05 November 2012
Accepted:	05 November 2012

Transaction Report:

(Note: With the exception of the correction of typographical or spelling errors that could be a source of ambiguity, letters and reports are not edited. The original formatting of letters and referee reports may not be reflected in this compilation.)

1st Editorial Decision 15 September 2012

Thank you for the submission of your manuscript "The exposure of autoantigens by microparticles underlies the formation of potent inflammatory components: the microparticle-associated immune complexes" to EMBO Molecular Medicine. We have now heard back from the three referees whom we asked to evaluate your manuscript. You will see that the referees find the topic of your manuscript potentially interesting. However, they also raise significant concerns on the study, which should be addressed in a major revision of the manuscript.

In particular, Reviewer #1 highlights that the data on the antigenicity of the particles, their ability to bind blood or synovial IgG and the immune activity of particles from synovial fluid should be extended.

Given the balance of these evaluations, we feel that we can consider a revision of your manuscript if you can convincingly address the issues that have been raised within the time constraints outlined below.

I look forward to seeing a revised form of your manuscript as soon as possible.

Yours sincerely,

Editor
EMBO Molecular Medicine

***** Reviewer's comments *****

Referee #1 (Comments on Novelty/Model System):

Previous studies have described the presence of platelet microparticles (MPs)in synovial fluid from patients with RA. The current studies utilize improved methodology and provide high quality data on the size of particles. The limitations relate to the model systems which involve creation of MP immune complexes and the generation of citrullinated proteins by PAD treatment. More interest in the findings would be generated by showing that the MPs from synovial fluid are a source of citrullinated proteins and that IgG from blood or synovial fluid can form complexes via binding to citrullinated or other antigens.

Referee #1 (Remarks):

In this manuscript, the authors extend findings of previous studies and present evidence that platelet microparticles (MPs) from synovial fluid (SF)can form immune complexes with immune activity.

1) The authors present data that MPs can be citrullinated to form immune complexes. Given that the MPs which come from synovial fluid (or blood) may have bound proteins that can be citrullinated, this may not be surprising. It would be important to show that MPs from SF have the citrullinated proteins rather than having proteins that can be citrullinated.
2) To show the presence of antigens on the MPs, the authors use what seem to be high concentrations of a goat anti-fibrinogen (250ug/ml) and a 1:100 dilution of anti-human vimenetin. Those seem like high concentrations and there is worry about IC binding in the preparations to Fc receptors. This should be explained. While the authors use IgG eluted from MPs, this could be in the form of bound immune complexes rather than antibodies binding to MP antigens themselves. Data in Figure 5 address these issues but they can be expanded and bolstered by additional controls.
3) To show the immunostimulatory activity of MP ICs, the authors use platelet MPs mixed with anti-fibrinogen. What about isolated MPs from synovial fluid or MPs mixed with serum from patients? Those experiments would provide more compelling evidence that the MPs can form complexes relevant to disease.
4) The current study focus on synovial fluid although a previous publication by another group indicated differences between synovial fluid and blood in the content of IgG positive particles. An important question therefore is whether there are differences in the MPs in SF vs. blood in antigenicity or whether the content of the relevant IgG differs in SF vs. blood perhaps because of local production. This issue should at least be discussed.
5) The anticoagulant for synovial fluid should be indicated.
6) It would be useful to specify clearly sources of MPs and platelets.

Referee #2 (Comments on Novelty/Model System):

In their manuscript W Robinson et al. demonstrate the presence of microparticle-containing immune complexes in RA. The findings of the manuscript are highly original and challange the old paradigm of immune complexes in rheumatology and autoimmunity research.
Because of the technical quality of the work, the study may also attract significant attention in the extracellular vesicle field.

Referee #2 (Remarks):

1. Both for the authors and EMBO Molecular Medicine high visibility of the work is of outmost importance. Therefore the authors are strongly recommended to use the synonym term "microvesicles" and the collective term "extracellular vesicle" in their manuscript (preferably in the abstract as well).
2. The authors should refer to the paper " Association of citrullinated proteins with synovial exosomes. Skriner K, Adolph K, Jungblut PR, Burmester GR. Arthritis Rheum. 2006 Dec;54(12):3809-14." that describes the presence of citrullinated autoantigens in RA synovial fluid extracellular vesicles.
3.Page 7. "Proteomic permits": please correct to "Proteomic analysis permits"
4. Do mPICs contain DNA in association with the detected histone H2A/a protein?

Referee #3:

The exposure of autoantigens by microparticles underlies the formation of potent inflammatory components: the microparticle-associated immune complexes
Cloutier N et al.

Immune complexes (IC) are pro-inflammatory macromolecular structures that exacerbate autoimmune diseases. Similar to ICs, submicron vesicles termed microparticles (MP) are present in the synovial fluid from patients affected with autoimmune arthritis and the authors examined the MPs in RA using high sensitivity flow cytometry and electron microscopy. They found that the MPs in RA synovial fluid are heterogeneous in size where the larger ones were MP-containing ICs; these accounted for the majority of the detectable ICs. The mpICs frequently expressed the platelet integrin CD41 suggesting a platelet origin and their expression of CD32 was not responsible for their formation. It appears that the mpICs display autoantigens such as vimentin and fibrinogen, and recognition of these targets by anticitrullinated peptide antibodies contributes to the production of mpICs. The platelet pICs were also highly pro-inflammatory, eliciting leukotriene production by neutrophils. The authors suggest a unique role for platelet MPs as autoantigen-expressing elements capable of perpetuating formation of inflammatory ICs.

Critique.

This was a well written manuscript that should have interest to the readership of EMBO Mol Med because it presents convincing evidence that platelet-derived MP's harbor IC and contain autoantigens that are recognized by patient autoantibodies.
The major criticism is related to the physical source of the MP; do the MP form in the SF or are they transported there from the vasculature. One way to test this is to examine peripheral blood platelets from the RA patients to determine their IC and autoantigen content.
The fonts of all the figures will never be seen in print. They should be enlarged.

1st Revision - authors' response 17 October 2012

Reviewer 1.

1. Previous studies have described the presence of platelet microparticles (MPs) in synovial fluid from patients with RA. The current studies utilize improved methodology and provide high quality data on the size of particles.

We are grateful to the reviewer's comments.

2. The limitations relate to the model systems which involve creation of MP immune complexes and the generation of citrullinated proteins by PAD treatment. More interest in the findings would be generated by showing that the MPs from synovial fluid are a source of citrullinated proteins and that IgG from blood or synovial fluid can form complexes via binding to citrullinated or other antigens.

In this new version of our manuscript, we have added important details on the antigenicity of the MPs. We examined the citrullination levels of the platelet MPs in the synovial fluid from RA patients. For comparison, we also evaluated the presence of citrullination on platelet MPs made *in vitro* and the platelet MPs contained in PA SF. Interestingly, only those from RA SF harbor citrullinations, pointing to a citrullination activity on platelet MPs that occurs in the course of RA. These results are now presented in the new figure 5A and B.

As for the PAD treatment of MPs (Figure 5C and D, former 5A and B), we would like underscore that these results don't simply indicate that platelet MPs are a substrate of PAD. Indeed, these observations also illustrate that the treatment of platelet MPs with PAD generates immune reactivity with autoantibodies from RA synovial fluid, and not those from PA synovial fluid. Thus, this clearly indicates that citrullinated proteins on platelet MPs can form complexes with IgG from RA patients. We believe this is an important concept.

Finally, we performed mass spectrometry on platelet MPs isolated from RA synovial fluid and surveyed the presence of RA canonical autoantigens. The autoantigens detected in platelet MPs are now presented in a new table (Table 1).

Remark 1.
The authors present data that MPs can be citrullinated to form immune complexes. Given that the MPs which come from synovial fluid (or blood) may have bound proteins that can be citrullinated, this may not be surprising. It would be important to show that MPs from SF have the citrullinated proteins rather than having proteins that can be citrullinated.

Novel figure 5A and B demonstrates that proteins in MPs from RA SF are indeed citrullinated (see also our comments above). Further, in the new Table 1 we demonstrate using mass spectrometry that platelet MPs comprise autoantigens. The result section takes these new observations in account pages 8-9.

New text page 8. Using hs-FCM, we first evaluated whether platelet MPs are citrullinated in the course of RA pathogenesis. We find that, unlike platelet MPs generated *in vitro* and those in PA SF, the platelet MPs in RA SF contain citrullinated epitopes (**Fig. 5A, B**), pointing to citrullination of MPs as a potential mechanism leading to recognition by autoantibodies.

New text page 9. Having identified autoantibodies in platelet mpICs, we next used mass spectrometry to determine whether platelet mpICs also include autoantigens. We demonstrate that several canonical RA autoantigens are present in platelet mpICs (**Table 1**).

Remark 2.
a) To show the presence of antigens on the MPs, the authors use what seem to be high concentrations of a goat anti-fibrinogen (250ug/ml) and a 1:100 dilution of anti-human vimentin. Those seem like high concentrations and there is worry about IC binding in the preparations to Fc receptors. This should be explained

We thank the reviewer for his careful examination of our manuscript. The anti-fibrinogen was used at 25 μg/ml (not 250 μg/ml) and we corrected this unfortunate typo. We took this opportunity to revisit all the details on methods and ensured their accuracy (no other details were missing). As for the anti-vimentin, we used it at the concentrations suggested by the manufacturer (estimation of the concentration is 25μg/ml). Furthermore, all the immuno-labelings were compared to their isotypic controls. Since all of these controls appeared negative, this rules out the role of Fc receptor in the formation of false positive. Finally, when the detection of MP surface antigens implicated usage of a combination of primary and secondary antibodies, the platelet Fc receptor CD32a was blocked with the Fab fragment IV.3 to abrogate the risk of artifactual binding of the antibodies to the Fc receptor.

Page 16. To detect vimentin and fibrinogen on surface of MPs, platelet MPs (20 X 10^6) and RA SF (20 μl) were pre-incubated with 20 μg/ml of CD32a blocking Fab fragment antibody for 15 minutes to abrogate any risk of potential non-specific binding of primary/secondary antibodies to platelet Fc receptor.

b) While the authors use IgG eluted from MPs, this could be in the form of bound immune complexes rather than antibodies binding to MP antigens themselves. Data in Figure 5 address these issues but they can be expanded and bolstered by additional controls.

We used an antigen Bioplex to determine what are the most abundant autoantibodies comprised in platelet mpICs. Considering that the majority of the immune complexes are in fact mpICs, these data are of high relevance. Like the reviewer mentioned, these IgG are either bound to MPs themselves or in the form on immune complexes and former Figure 5 (new Figure 6) addresses this issue. We believe that our new data, added to those presented in our former version of the manuscript, convincingly demonstrate that autoantibodies can bind MPs directly.

Indeed, we show that

1. Incubation of platelets MPs in RA SF, not PA SF, promotes generations of mpICs (thus the IgG bind MPs) (Figure 4D-F).
2. Platelet MPs in RA, and not those *in vitro* nor from PA, are citrullinated (Figure 5B).
3. Citrullination of platelet MPs generates immuno-reactivity to autoantibodies from RA SF (thus MPs are recognized by IgG) (Figure 5C).

4. Autoantigens are detected by mass spectrometry in mpICs (new Table 1) and are detected on surface of platelet MPs (thus IgG bind MPs) (Figure 6B-D).

Together, our results evidence and suffice to establish that in RA, MPs expose autoantigens leading to formation of active MP-containing immune complexes.

Remark 3.
To show the immunostimulatory activity of MP ICs, the authors use platelet MPs mixed with anti-fibrinogen. What about isolated MPs from synovial fluid or MPs mixed with serum from patients? Those experiments would provide more compelling evidence that the MPs can form complexes relevant to disease.

We used mpICs isolated from RA SF to stimulate neutrophils. We present these data in the new Figure 7A.

Remark 4.
The current study focus on synovial fluid although a previous publication by another group indicated differences between synovial fluid and blood in the content of IgG positive particles. An important question therefore is whether there are differences in the MPs in SF vs. blood in antigenicity or whether the content of the relevant IgG differs in SF vs. blood perhaps because of local production. This issue should at least be discussed.

This topic is now discussed in this version of the manuscript.

New text page 11. The presence of immunoglobulins on surface of MPs in blood during RA has been previously examined (Nielsen et al, 2012; van Eijk et al, 2010). In contrast to our demonstration of MP interactions with ICs in RA SF, those studies did not find evidence of MP association with ICs in RA blood. Although these observations may point to the existence of highly potent vascular and reticular-endothelial system clearance mechanisms, this may also suggest that the mpICs form locally, inside the inflamed joint in the SF. Indeed, the MPs derived by platelets in blood during RA (Knijff-Dutmer et al, 2002; Sellam et al, 2009) may accumulate in the SF through the gaps present between the endothelial cells in the inflamed vasculature (Boilard et al, 2012; Cloutier et al, 2012). Once in SF (where PAD4 is present), MPs may undergo citrullination and form mpICs. The exact anatomical site where mpICs form and the mechanism(s) of transportation of mpICs remains the subject of intense investigation.

Remark 5.
The anticoagulant for synovial fluid should be indicated.

No anticoagulant was used. It is now specified in the methods.

Page 14. Fresh SF harvested without anticoagulant were cleared of leukocytes by centrifugation at 1,900g for 30min at 4°C, were aliquoted and were kept at -80 ^{o}C.

Remark 6.
It would be useful to specify clearly sources of MPs and platelets.

We ensured that the sources are always well specified.

Reviewer 2.

In their manuscript W Robinson et al. demonstrate the presence of microparticle-containing immune complexes in RA. The findings of the manuscript are highly original and challenge the old paradigm of immune complexes in rheumatology and autoimmunity research. Because of the technical quality of the work, the study may also attract significant attention in the extracellular vesicle field.

Remark 1.
Both for the authors and EMBO Molecular Medicine high visibility of the work is of outmost importance. Therefore the authors are strongly recommended to use the synonym term "microvesicles" and the collective term "extracellular vesicle" in their manuscript (preferably in the abstract as well).

We included these terms in the introduction, keywords and abstract.

Remark 2.
The authors should refer to the paper " Association of citrullinated proteins with synovial exosomes. Skriner K, Adolph K, Jungblut PR, Burmester GR. Arthritis Rheum. 2006 Dec;54(12):3809-14." that describes the presence of citrullinated autoantigens in RA synovial fluid extracellular vesicles.

We mention/cite this paper in the new version of our manuscript.

New text page 12. Other types of extracellular vesicles may express autoantigens. Small apoptotic $CD3^+$ T-cells, for instance, expose vimentin (Boilard et al, 2003) and could potentially interact with anti-vimentin autoantibodies if present in RA SF. Furthermore, exosomes, vesicles derived from intracellular compartments, also display antigens in RA SF (Skriner et al, 2006). MPs from other sources (other than platelet-derived) may also express autoantigens. Considering that MPs from multiple sources populate the SF (Berckmans et al, 2002; Boilard et al, 2010), it is highly plausible that they too form mpICs. We revealed the capability of PAD4 to use platelet MPs as substrate. This process is likely to occur on other MPs, regardless of their origin, promoting the formation of additional citrullinated autoantigens in RA SF. However their identity and contribution remain to be established.

Remark 3.
Page 7. "Proteomic permits": please correct to "Proteomic analysis permits"

We made the correction.

Page 8. Proteomic analyses permit the identification of the antigens targeted by autoantibodies.

Remark 4.
Do mPICs contain DNA in association with the detected histone H2A/a protein?

This is an interesting question. Indeed we detect both histones and anti-histone antibodies in mpICs. Interestingly, histones by themselves are able to bind platelets and to promote their activation. Indeed, the injection of histones in mice can even promote thrombocytopenia. Histones may thus be bound directly to MPs derived from platelets, or indirectly via DNA. Although this is the topic of active investigation, we mention these possibilities in the new discussion.

New text page 12. Among these other proteins, only histones were also identified in our mass spectrometry analyses. Histones may be associated with DNA, itself bound to MPs. Alternatively, histones are recognized potent platelet stimuli and can associate with platelets directly (Fuchs et al, 2011). Platelet MPs, like platelets, may thus interact with histones, which once citrullinated, could be recognized by anti-histone autoantibodies. Although the contribution of these autoantigens to mpIC formation remains to be identified, we establish that major autoantigens in RA may arise from the surface of platelet-derived MPs.

Reviewer 3.

1. This was a well written manuscript that should have interest to the readership of EMBO Mol Med because it presents convincing evidence that platelet-derived MP's harbor IC and contain autoantigens that are recognized by patient autoantibodies.

We thank the reviewer for his comments.

2. The major criticism is related to the physical source of the MP; do the MP form in the SF or are they transported there from the vasculature. One way to test this is to examine peripheral blood platelets from the RA patients to determine their IC and autoantigen content.

We discuss these issues in the new version of the manuscript.

New text page 11. The presence of immunoglobulins on surface of MPs in blood during RA has been previously examined (Nielsen et al, 2012; van Eijk et al, 2010). In contrast to our demonstration of MP

interactions with ICs in RA SF, those studies did not find evidence of MP association with ICs in RA blood. Although these observations may point to the existence of highly potent vascular and reticular-endothelial system clearance mechanisms, this may also suggest that the mpICs form locally, inside the inflamed joint in the SF. Indeed, the MPs derived by platelets in blood during RA (Knijff-Dutmer et al, 2002; Sellam et al, 2009) may accumulate in the SF through the gaps present between the endothelial cells in the inflamed vasculature (Boilard et al, 2012; Cloutier et al, 2012). Once in SF (where PAD4 is present), MPs may undergo citrullination and form mpICs. The exact anatomical site where mpICs form and the mechanism(s) of transportation of mpICs remains the subject of intense investigation.

3. The fonts of all the figures will never be seen in print. They should be enlarged.

We have enlarged all the figures and ensured they showed well once printed.

2nd Editorial Decision 05 November 2012

Please find enclosed the final reports on your manuscript. We are pleased to inform you that your manuscript is accepted for publication.

Congratulations on your interesting work,

Sincerely yours,

Editor
EMBO Molecular Medicine

***** Reviewer's comments *****

Referee #1 (Comments on Novelty/Model System):

The revisions address concerns in the original review. The models are appropriate. There are no ethical issues.

Referee #1 (Remarks):

The revisions address concerns in the original review.

Referee #3 (Comments on Novelty/Model System):

The authors have adequately addressed my comments. The paper is much improved and ready for publication.

Referee #3 (Remarks):

The authors have adequately addressed my comments.
