# Supplementary material for: The exposure of autoantigens by microparticles underlies the formation of potent inflammatory components: the microparticle-associated immune complexes
Source: EMBO Mol Med. 2012 Dec 11;5(2):235–49. doi: 10.1002/emmm.201201846 (PMC3569640; doi:10.1002/emmm.201201846)
Supplement: Supplementary file 2 [file emmm0005-0235-SD2.pdf]

Supplementary material

The exposure of autoantigens by microparticles underlies the formation of potent inflammatory components: the microparticle-associated immune complexes

Nathalie Cloutier[1], Sisareuth Tan[2], Luc H. Boudreau[1], Catriona Cramb[3], Roopashree Subbaiah[4], Lauren Lahey[3], Alexandra Albert[1], Ruslan Shnayder[5], Reuben Gobezie[6], Peter A. Nigrovic[5,7], Richard W. Farndale[8], William H. Robinson[3], Alain Brisson[2], David M. Lee[5,9] and Eric Boilard[1]

Table of contents

Supplementary Figure 1 2
Supplementary Figure 2 3
Supplementary Table 1 4
Supplementary Table 2 5

Supplementary Figure 1

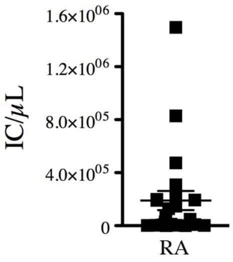

Supplementary Figure 1. Quantification of ICs detected in RA SF. Flow cytometric quantifications of the total IgG^+ ICs in RA SF (n = 23). Data are mean ± SEM.

Supplementary Figure 2

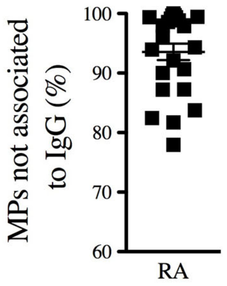

Supplementary Figure 2. Quantification of the MPs not associated to IgG in RA SF. Flow cytometric quantifications of the Annexin-V^+ MPs not associated to IgG in RA SF (n = 23). Data are mean ± SEM.

Supplementary Table 1. List of antigens evaluated by Bio-PlexTM bead based antigen array

Apolipoprotein A1	Fibrinogen A (556-575 cit sm cyclic)
Apolipoprotein A1 cit	Fibrinogen A (616-635 cit3 cyclic)
Apolipoprotein E	Fibrinogen A (616-635 cit3 small cyclic)
Apolipoprotein E cit	Fibrinogen A1 (616-635 cit3)
Apolipoprotein E (277-296 cit2 sm1 cyclic)	Fibrinogen A2 (41-60 cit3)
Apolipoprotein E (277-296 cit2 sm2 cyclic)	Fibrinogen A10 (556-575 cit)
Biglycan (247-266 cit sm-1 cyclic)	Fibrinogen B (60-74 cit cyclic small-2)
CCP	Filaggrin (48-75 cit2v2 cyclic)
Cfc (48-65 cit cyclic)	Filaggrin (48-65 cit2 v1 cyclic)
Cfc (48-65 cit1)	Filaggrin 1 (48-65 cit2v1)
Clusterin (221-240 cit cyclic)	Filaggrin 2 (48-65 cit2v2)
Clusterin (231-250 cit sm-1 cyclic)	H2A/a (1-20 cit sm-2 cyclic)
Clusterin (231-250 cit sm-2 cyclic)	H2A/a-2 (1-20 cit)
Clusterin-5 (231-250 cit)	H2B/a (62-81 cit cyclic)
Collagen pC11 (Cit1 cyclic)	Histones 2B
Enolase 1A (5-21 cit)	Histones 2B cit
Enolase 1A (cyclic)	HSP60
Enolase 1B (5-21 cit)	Vimentin
Fibrinogen A	Vimentin cit
Fibrinogen A cit	Vimentin (58-77 cit3 cyclic small-1)

Supplementary Table 2. List of the RA canonical antigens evaluated by mass spectrometry

Apolipoprotein A1	Fibrinogen A2
Apolipoprotein E	Fibrinogen A10
Biglycan	Fibrinogen B
CCP	Filaggrin
Cfc	Filaggrin 1
Clusterin	Filaggrin 2
Clusterin-5	Histone 2A/a
Collagen pC11	Histone 2A/a-2
Enolase 1A	Histone 2B/a
Enolase 1B	Histone 2B
Fibrinogen A	HSP60
Fibrinogen A1	Vimentin
